# Polyphenols and IUGR Pregnancies: Effects of the Antioxidant Hydroxytyrosol on the Hippocampus Proteome in a Porcine Model

**DOI:** 10.3390/antiox11061135

**Published:** 2022-06-09

**Authors:** Natalia Yeste, Jorge Pérez-Valle, Marta Vázquez-Gómez, Consolación García-Contreras, Antonio González-Bulnes, Anna Bassols

**Affiliations:** 1 Departament de Bioquímica i Biologia Molecular, Facultat de Veterinària, Universitat Autònoma de Barcelona, Cerdanyola del Vallès, 08193 Barcelona, Spain; natalia.yeste@uab.cat (N.Y.); jorge.perez.valle@uab.cat (J.P.-V.); 2 Faculty of Veterinary Sciences, UCM, Ciudad Universitaria s/n, 28040 Madrid, Spain; mvgomez@ucm.es (M.V.-G.); antonio.gonzalezbulnes@uchceu.es (A.G.-B.); 3 Nutrition et Obesités: Approaches Systèmiques, NutriOmique, Sorbonne Université, INSERM, 75013 Paris, France; 4 Department of Nutrition and Sustainable Animal Production, Estacion Experimental del Zaidin, CSIC, 18008 Granada, Spain; consolacion.garcia@eez.csic.es; 5 Comparative Physiology Group, INIA, Avda. Puerta de Hierro s/n, 28040 Madrid, Spain; 6 Departamento de Producción y Sanidad Animal, Facultad de Veterinaria, Universidad Cardenal Herrera-CEU, CEU Universities, Tirant lo Blanc, 7, Alfara del Patriarca, 46115 Valencia, Spain

**Keywords:** hydroxytyrosol, hippocampus, intrauterine growth restriction, brain, pig, proteome, TMT labelling

## Abstract

Supplementation of a mother’s diet with antioxidants such as hydroxytyrosol (HTX) has been proposed to ameliorate the adverse phenotypes of foetuses affected by intrauterine growth restriction (IUGR). Our previous studies showed, in a porcine model of IUGR, an effect of maternal HTX supplementation on the neurotransmitter profile of several brain areas and the morphology of the hippocampus in 100 days old foetuses. The present study analyzed the impact of maternal HTX supplementation on the hippocampus proteome at this foetal age by TMT10plex labelling. Eleven differentially abundant proteins were identified by comparing both conditions, and eight of them downregulated and three upregulated in the HTX-treated group. The downregulated proteins were mainly involved in protein synthesis and RNA metabolism and may explain the differences in neuron differentiation in the HTX-treated group. The upregulated proteins were related to cell detoxification and could represent a potential mechanism to explain the neuroprotective effect of HTX.

## 1. Introduction

Intrauterine growth restriction (IUGR) leads to low birthweight offspring [1,2]. In humans, IUGR is associated with an increased risk of perinatal mortality, and surviving offspring are predisposed to chronic disorders such as obesity, type II diabetes, and cardiovascular diseases [3,4].

The nutritional prevention of the deleterious consequences of IUGR is an open novel approach to confronting disease during the foetal period, especially due to the problems associated with pharmaceutical intervention during pregnancy. The importance of diet during pregnancy has been extensively demonstrated. Special attention has been given to the Mediterranean diet, which is based on virgin olive oil and vegetables and fruits, and is rich in antioxidants such as polyphenols [5,6]. Hydroxytyrosol (HTX), abundant in virgin olive oil, has antioxidant, anti-inflammatory, and immunomodulatory activities that make this polyphenol especially interesting [7,8]. Furthermore, it is easily absorbed in the intestine, transported in the plasma [9], and able to cross the blood–brain barrier [10]. In the nervous system, HTX and other polyphenols have neuroprotective effects since they reduce the overall oxidative damage associated with their antioxidant effect and the improvement of mitochondrial function [7,11,12,13,14].

Oxidative stress is an important issue during gestation, especially in pathologic pregnancies and at certain periods of placental development [15]. For this reason, the efficacy of antioxidants (e.g., antioxidant vitamins and melatonin) to ameliorate adverse phenotypes has been investigated in humans [16,17] and animals [18,19,20]. There are indications that maternal HTX administration may improve neurogenesis and cognitive function in the offspring of prenatally stressed rats [11].

Our groups have developed a porcine model for IUGR by limiting the caloric intake of the mothers to 50% of the daily maintenance requirements from gestational day 35, which is around one-third of the pregnancy length [21,22]. Previous studies of ours [23,24,25] and of other groups [26,27,28,29,30,31,32,33] have described that IUGR affects the central nervous system, in addition to being detrimental to growth and metabolism.

Using this porcine model, the previous studies of our research groups determined that maternal HTX supplementation in pregnancies at risk of IUGR improved the growth of the offspring during prenatal and postnatal periods and had benefits on their metabolic traits [34,35,36,37]. Moreover, HTX maternal supplementation altered the neurotransmitter profile in several brain areas and in neuron differentiation in the hippocampus in 100 day old foetuses. In that study, the effects of HTX were observed only in foetuses, whereas there were no differences in 1 month and 6 month old pigs between the control and HTX-supplemented groups, suggesting that the continuous presence of HTX was needed to maintain its protective effects on the brain [38].

Based on these previous results, the aims of the present study were to extend these results by examining the effects of maternal HTX supplementation on the proteome of the hippocampus, a brain area involved in complex functions (memory, learning, mood, emotion, stress, cognition, etc.), with the goal of identifying potential mechanisms for the neuroprotective effects of this compound.

## 2. Materials and Methods

### 2.1. Animals and Experimental Procedure and Ethics Statement

The study involved 13 purebred Iberian sows in their third parity. The treatment and management of sows, as well as the collection of foetuses and foetal samples, were as described in our previous work [38,39]. Briefly, sows were fed with a standard diet up to day 35 of gestation, and at this time, the food amount was adjusted to fulfil 50% of their daily requirements. At day 35, sows were pair-matched according to body weight and 7 females became the untreated control group (Ctrl), whilst the 6 remaining females became the treated group (HTX, mothers supplemented with 1.5 mg HTX/kg feed) until day 100 of pregnancy, when the foetuses were obtained.

For the proteomic studies described here, the same individuals that were analysed by immunohistochemistry in our previous work were used [39]. Only foetuses with normal body weight (NBW) were selected to reduce the number of variables and because the number of available individuals was higher in the NBW group. Twenty individuals were chosen, ten for each group (Ctrl and HTX) and sex (male or female). This means that we had five CF (control-female) individuals, five CM (control-male) individuals, five TF (treated-female) individuals, and five TM (treated-male) individuals, as indicated in Table 1. The Ctrl group was obtained from five different litters (one male and one female for each litter) and the HTX group from three different litters, making pairs of males females per litter, where possible. The subjects’ body weights were 822.1 ± 29.3 g for the Ctrl group and 817.0 ± 24.8 g for the HTX group.

Sampling was performed after stunning and exsanguination, in compliance with RD53/2013 standard procedures. Foetuses were obtained at day 100 of pregnancy, sexed, and weighed immediately after retrieval. The brain was removed from the skull and the hippocampi were dissected [38,39]. The study was performed according to the Spanish Policy for Animal Protection RD53/2013, which meets the European Union Directive 2010/63/UE about the protection of animals used in research. The experiment was assessed and approved (report CEEA 2013/036) by the INIA Committee of Ethics in Animal Research, which is the named Institutional Animal Care and Use Committee (IACUC) for the INIA. The sows were housed at the animal facilities of the INIA, which meets the local, national, and European requirements for Scientific Procedure Establishments.

### 2.2. Isobaric Mass Tag Labelling with TMT10plex™

Protein extracts were prepared by sonication (30% amplitude, 1 s × 10) in 150 mM NaCl, 50 mM Tris-HCl of pH 7.5, and 1% NP-40 plus a cocktail of protease inhibitors and followed with centrifugation. Protein quantification was performed using the microBCA method (Thermo Scientific, Waltham, MA, USA, #23235). Trypsin digestion was performed following the FASP protocol and digestion performance control was conducted by spotting 0.5 µL of the eluted peptides from each sample onto a MALDI plate by the dried droplet method and analysing at a range of 800 to 4000 *m/z* in a 4800 AB-Sciex MALDI-TOF/TOF MS.

Peptides were evaporated in speed-vac, and the evaporated tryptic peptide samples were reconstituted with 100 µL 20 mM TEAB (triethylammonium bicarbonate buffer; Sigma, St. Louis, MO, USA, # T7408) and labelled with TMT10plex isobaric label reagents (ThermoFisher, #15285743). The experimental design for the TMT10 labelling is shown in Table 1.

### 2.3. High pH-Reverse Phase (HP-RP) Liquid Chromatography and LC-MS/MS Analysis

Each labelling reaction was quenched with 5% hydroxylamine for 15 min before each batch was mixed into a single TMT 10-plex tube. Labelled peptide mixes were cleaned-up in C18 cartridges (Agilent Technologies, Santa Clara, CA, USA, #A5320320). Each 10-plex mix (corresponding to ~600 µg initial protein) was reconstituted with 300 µL 5 mM ammonium formate, pH 10, 2% ACN, to create a 2 µg/µL solution. Two hundred µL of each mix (~400 µg) was fractionated in a HPLC 1100 UV-Vis (Agilent) with an XBridge Peptide BEH C18, 130 Å, 5 µm, 2.1 mm × 100 mm column (Waters, Milford, MA, USA, #186003575).

The HP-RP chromatographic gradient started at 97% solvent A (5 mM ammonium formate, pH 10, 2% ACN) and 3% solvent B (5 mM ammonium formate, pH 10, 90% ACN) at 200 µL/min flow for a total run time of 76 min. Fractions were collected every 2 min (400 µL) and maintained at −40 °C. In accordance with the chromatographic profiles obtained, fractions from 26 to 60 min were considered for further analysis. Six mixes of 40 µL of three different fractions were conducted for each labelled mix. Aliquots of 40 µL of these fraction mixes were frozen and evaporated in a speed-vac before LC-MS/MS analysis.

High-resolution LC-MS/MS was conducted for the 12 fraction mixes. The MS system used was a Fusion Lumos™ Tribrid mass spectrometer (Thermo Scientific, Waltham, MA, USA) coupled to a Thermo Scientific Dionex Ultimate 3000 chromatographic system. The equivalent to 500 nanograms from each fraction was loaded into an Acclaim PepMap100 C18 Trap column (100 μm × 2 cm, 5 μm, 100 Å; Thermo Scientific) at 15 μL/min, connected to a NanoEase MZ HSS T3 column (75 μm × 250 cm, 1.8 μm, 100 Å; Waters). The separation was done at 250 nL/min with a gradient 3–35% ACN (0.1% FA) in 60 min. The end of the chromatographic column was directly connected to an Advion TriVersa NanoMate system coupled to the spectrometer, working at 1.7 kV.

The spectrometric analysis was performed in a data-dependent mode, *m/z* range 375–1500, SPS3 fragmentation, top speed method, with a collision energy of 35% for MS2 CID and 65% for MS3 HCD. The detection was done in the Orbitrap (MS1 120k), Ion trap (MS2), and Orbitrap (MS3 60 kDa). The software for instrument acquisition was Xcalibur v 4.2.28.14 (Thermo Scientific).

### 2.4. Data Analysis and Quantification

The identification of the peptides in the database was carried out using Proteome Discoverer v2.5 software (Thermo Scientific, Waltham, MA, USA) using a 1% FDR and the Uniprot database restricted to *Sus scrofa* taxonomy (released 2021/02) and contaminants (released 2017). The enzyme was set to trypsin with two missed cleavages allowed. The dynamic modifications were oxidation in methionine, acetylation in protein *N*-terminus, Met-loss in methionine, and Met-loss+Acetyl in methionine. The carbamidomethylation of cysteines and the TMT in peptide N-terminus and in K were set as the static modifications. The precursor and fragment mass tolerance were set to 100 ppm and 0.6 Da.

Quantification of the proteins was based on the intensity of the reporter ions derived from the TMT labelling. Only unique peptides with SPS mass matches higher than 55% were used for quantification. The TMT intensity of the reporter ions was normalized using the total abundance of each TMT label to minimize the error due to the different protein load of each channel. Statistical analysis to determine the differential proteins and peptides was performed using DanteR software (http://omics.pnl.gov/software/danter accessed on 14 July 2021). Detailed information on how this software works can be found at http://omics.pnl.gov/sites/defaμlt/files/DanteR_Overview.pdf (accessed on 14 July 2021). An ANOVA analysis of control versus treated samples using the normalized data has been performed using DanteR software. The variability introduced by the labelling and by the sex of the animals from which the samples were obtained is included in the linear model. *P*-values were adjusted using the Benjamini and Hochberg correction.

### 2.5. Gene Ontology and Bioinformatic Analysis

For protein names and gene ontology (GO) classifications, PANTHER version 16.0 software (http://pantherdb.org/ accessed on 22 September 2021) was used, together with the UniProt databases (http://www.uniprot.org/ accessed on 22 September 2021) [40]. Complete GO and GO slims were run. GO slims are cut-down versions of the GO ontologies containing a subset of the terms in the whole GO. They give a broad overview of the ontology content but exclude the details of the specific fine-grained terms. For pathway analysis, the Reactome platform version 77 was used (https://reactome.org/ accessed on 23 September 2021) [41], as well as the Kegg Mapper tool version 5.0 (https://www.genome.jp/kegg/mapper.html accessed on 23 September 2021) [42]. For protein interaction network analyses, the identified proteins were analysed with STRING version 11.5 (http://string-db.org/ accessed on 30 March 2022).

The MS proteomics data has been uploaded to the ProteomeXchange Consortium via the PRIDE partner repository [43] with the dataset identifier PXD033684 (Username: reviewer_pxd033684@ebi.ac.uk; Password: DIjoO0vY).

## 3. Results

### 3.1. Effects of Maternal Supplementation with HTX on the Foetal Hippocampal Proteome

To identify the proteins differentially expressed in the hippocampus between the two study conditions, twenty samples were used: ten of each condition (Ctrl and HTX) and sex (M and F). The samples were from the same foetuses used in the previous study [39].

The identification of the peptides in the database was carried out using the Proteome Discoverer v2.5 software using a 1% FDR and the Uniprot database restricted to the taxonomy and contaminants of *Sus scrofa*. A total of 5745 proteins were identified, 4479 with at least 2 peptides. An ANOVA analysis of the control (Ctrl) versus the treated (HTX) samples was performed using the normalized data, and an FC of > 1.5 (upregulated) or an FC of < 0.67 (downregulated) and a *p*-value of less than 0.05 were established as the selection criteria. Following these thresholds, only 11 differentially abundant proteins (DAP) were identified (Table 2). Three proteins were more abundant in the group whose mothers had been supplemented with HTX, and eight proteins were decreased. There was no significant effect of sex in this analysis.

### 3.2. Gene Ontology Analysis

**Figure 1:**
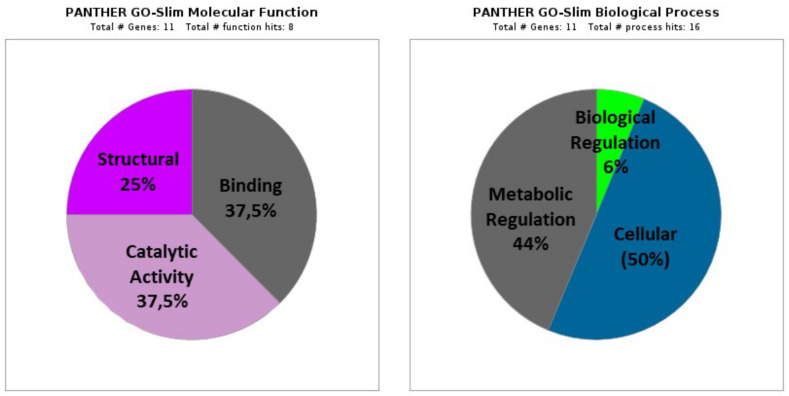
GO-slim categories of DAPs in the comparison between the Ctrl and HTX groups.

The GO categories of the 11 DAPs based on molecular function and biological processes are depicted in Figure 1 and are summarized as follows:

#### 3.2.1. Molecular Function

(A) Binding proteins (GO: 0005488; 37.5%), including three proteins that represent several functions: ion binding (GO:0043167; one protein: HSPE1), heterocyclic compounds binding (GO:1901363; two proteins: RPL7A and RPL7), organic compounds binding (GO:0097159; two proteins: RPL7A and RPL7), and other proteins binding (GO:0005515; one protein: HSPE1).
(B) Proteins with catalytic activity (GO:0003824; 37.5%), including three proteins classified as ligases (GO:0016874; one protein: TARS1), lyases (GO:0016829; two proteins: HADHA and ECHDC1), on RNA (GO:0140098; one protein: TARS1), and oxidoreductases (GO:0016491; one protein: HADHA).
(C) Structural Proteins (GO:0005198; 25%), including two proteins corresponding to structural components of the ribosome (GO:0003735; RPL36 and RPL7).

#### 3.2.2. Biological Process

(A) Cellular processes (GO: 0009987; 50%), including eight proteins corresponding mainly to metabolism (GO: 0044237; seven proteins: RPL36, RPL7A, RPL7, TARS1, HADHA, ECHDC1, and RBMX), protein folding (GO:0006457; one protein: HSPE1), and cellular component organization or biogenesis (GO:0071840; two proteins: RPL7A and RPL7).
(B) Metabolic Processes (GO: 0008152; 44%), including seven proteins (RPL36, RPL7A, RPL7, TARS1, HADHA, ECHDC1, and RBMX) that mainly participate in the metabolism of organic compounds (GO: 0071704), cellular metabolism (GO:0044237), and primary metabolism (GO:0044238).
(C) Regulation of biological processes (GO:0065007; 6%) with one protein (RBMX).

The results of the GO statistical overrepresentation test are shown in Supplementary Table S1, with an FC of between 8 and > 100 and *p*-values of between 1.7 × 10^−5^ and 2.3 × 10^−7^.

### 3.3. Pathway Analysis with Reactome and KEGG

The analysis of the pathways using Reactome showed that the main nodes were “Metabolism” (specifically, the β-oxidation of fatty acids in the mitochondria), “Protein metabolism” (mostly pathways related to protein translation and folding), “RNA metabolism”, “Signal transduction”, and “Developmental biology” (especially axon guidance during the development of the nervous system).

In the analysis with KEGG, ribosome and gene translation pathways were found to be prominent, with four of the differential proteins involved; metabolic pathways with two proteins (involved in fatty acid and amino acid metabolism); aminoacyl-tRNA biosynthesis (one protein); spliceosome (one protein); and protein processing in the endoplasmic reticulum (one protein).

### 3.4. Network Analysis with String

**Figure 2:**
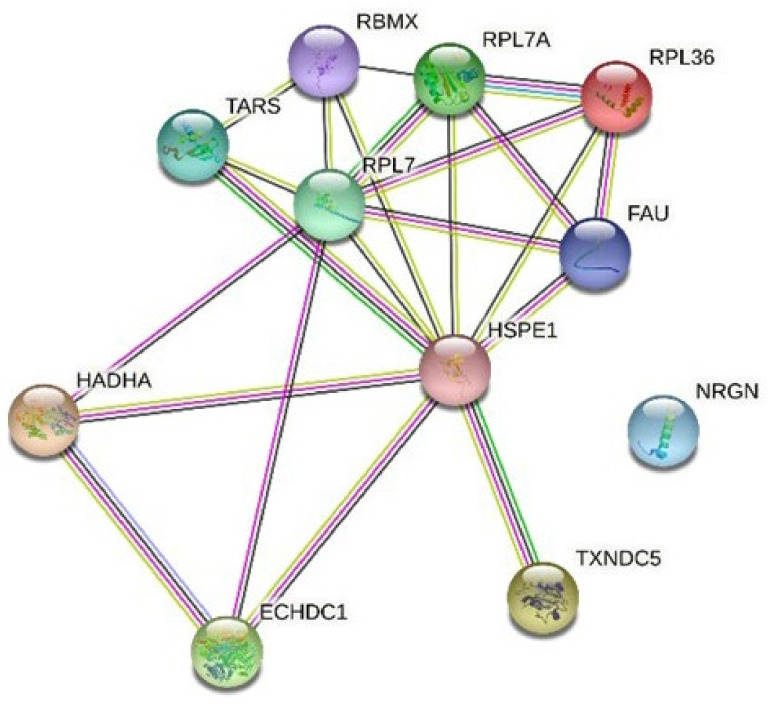
Graphic representation by STRING of the interactions of the identified DAPs. The nodes represent the identified proteins, and the lines represent associations from the different sources: blue from databases, pink from experimental determinations, dark green from predictions due to closeness of genes, yellow from to appearance in documents, and black from co-expression.

Finally, for the graphic visualization of these interactions, the STRING tool was used and included the predictions of interactions between proteins. The analysis showed the existence of a main node composed of ribosomal proteins (Figure 2).

## 4. Discussion

The present work intends to delve into the molecular mechanisms that may explain the beneficial effects of HTX on the brain. Proteomic approaches offer a quantitative, high throughput methodology to assess the changes in protein composition, and our groups have previously used these methodologies to characterize proteomic changes in the porcine hippocampus [25,44]. The results obtained here indicate that HTX produced few changes in the hippocampal proteome, since only eleven DAPs were identified between foetuses from the control and HTX-supplemented mothers. This result indicates that HTX probably modifies the redox state of cells but does not substantially change the proteins that constitute cells.

Of the eleven DAPs identified, three of them (ECHDC1, TXNDC5, and NRGN) were more abundant in foetuses whose mothers had been supplemented with HTX. ECHDC1 is the enzyme ethylmalonyl-CoA decarboxylase and is responsible for decarboxylating ethylmalonyl-CoA, a potentially toxic metabolite, to form butyryl-CoA [45]. Ethylmalonyl-CoA may be formed as a by-product of acetyl-CoA carboxylase and may be incorporated as ethyl-branched fatty acids into different lipids, including phosphatidylcholines and plasmanylcholines [46], which are abundant in the nervous system and other tissues. TXNDC5 is a thioredoxin, which is an endoplasmic reticulum protein. Due to its disulphide isomerase activity, TXNDC5 reduces the incorrect disulphide bonds formed in the newly folded proteins and then catalyses the oxidation of residues to arrange disulphide bonds in the native structure. Besides, TXNDC5 has a chaperone activity and promotes the correct protein folding in the ER [47].

Neurogranin (Ng) is expressed in dendritic spines in the brain and acts as a messenger in protein kinase C signalling cascades in the synapses [48]. There is a clear correlation between Ng levels and the cognitive function in, for example, aging and hypothyroidism. Mice deficient in Ng show severe deficits in visual–spatial learning and a marked tendency towards stress and anxiety [49]. The increase in Ng in the HTX group may be related to the IHC results [38,39], which indicate that HTX maternal supplementation induces more mature neurons. Therefore, it may be translated into greater synaptic activity. In summary, the proteins that were increased by HTX supplementation have a detoxifying and protective effect on cells and are involved in synapse formation, suggesting a potential mechanism for the neuroprotective effects of HTX.

Eight DAPs were decreased in foetuses whose mothers were supplemented with HTX, (HSPE1, TARS1, FAU, RPL7A, RPL36, RPL7, HADHA, and RBMX). The main category was composed of four ribosomal proteins: FAU (40S ribosomal protein S30), RPL7A, RPL36, and RPL7, which are part of the 40S and 60S subunits. Since ribosomal proteins are involved in protein synthesis, a decrease in these proteins suggests that protein synthesis may work at a slower rate in the hippocampus of the HTX group. Since it appears that, in this condition, cell differentiation prevails over-proliferation [38], this may explain the decrease. It can be speculated that, if this effect was to be translated to other tissues and organs, it could explain why HTX foetuses had lower weight than CTRL foetuses. In previous works from our groups, Vázquez-Gómez et al. evaluated the relative weights of different organs and reported that animals in the HTX group had lower head and brain weights compared to the Ctrl group from weaning up to 60 days. After 60 days, the HTX group had a higher body weight than the Ctrl group [34,35].

HSPE1 is a heat shock protein involved in mitochondrial protein import and macromolecular assembly. Furthermore, it prevents misfolding and promotes proper refolding and assembly under stress conditions in the mitochondrial matrix [50]. This fact may be linked to decreased protein synthesis and less need for error correction mechanisms. Other proteins found to be decreased in animals supplemented with HTX were: TARS1, an enzyme that catalyses the binding of the amino acid threonine to tRNA [51]; and RBMX, which is an RNA-binding protein that regulates pre- and post-transcriptional processes and is also part of the spliceosome, regulating mRNA splicing [52,53]. These two proteins also are related to protein metabolism and synthesis, which could agree with the decrease in ribosomal proteins.

HADHA is part of an enzyme complex called mitochondrial trifunctional protein, which is essential for the oxidation of long-chain fatty acids as it catalyses the last three steps of beta-oxidation [54]. It is possible to speculate that the decrease in protein synthesis implies a lower need for energy, and therefore a lower degradation of fatty acids, even if it is well recognized that the brain has a minor utilization of long-chain fatty acids to obtain energy [55].

The effect of sex was not relevant, similar to our previous studies on the impact of HTX on brain NTs and hippocampal development [38]. In contrast, there was an influence of sex on the effects of HTX on plasma metabolic markers and antioxidant status [36].

## 5. Conclusions

In conclusion, although there is plenty of information about the neuroprotective effects of HTX, there is a paucity of data about the underlying mechanisms, especially in risk-prone pregnancies. Our results are focused on the hippocampus, given the importance of this brain area, and offer potential explanations for the effects of maternal supplementation with HTX on an offspring’s brain. Furthermore, the usefulness of proteomic approaches to envision biological mechanisms is emphasized.

## Figures and Tables

**Table 1:** Experimental design for the TMT10-plex labelling. Two experiments and ten labelling reactions were performed for a total of twenty samples randomly distributed to avoid labelling bias. Samples are identified as C (Ctrl), T (treated with HTX), sex (male M, female F), and number of the individual.

	Reporter									
	126	127N	127C	128N	128C	129N	129C	130N	130C	131
Experiment 1	CF.1	CM.5	CF.2	TM.1	TF.5	CF.3	CM.4	TF.4	TM.2	TF.3
Experiment 2	CM.3	TF.2	TM.3	CF.4	CM.2	TM.4	TF.1	CM.1	CF.5	TM.5

**Table 2:** Differentially abundant proteins between the Ctrl and HTX groups identified after TMT10-plex labelling. FC > 1.00 refers to an increase in HTX, and FC < 1.00 refers to a decrease in HTX.

Access Uniprot	Gen	Identification	FC	*p*-Value
F1SMZ6	*HSPE1*	10 kDa heat shock protein, mitochondrial	0.06	0.048
F1SP18	*TARS1*	Threonyl-tRNA synthetase	0.50	0.019
P62863	*FAU*	40S ribosomal protein S30	0.52	0.002
Q29375	*RPL7A*	60S ribosomal protein L7a	0.56	0.007
Q29554	*HADHA*	Trifunctional enzyme subunit alpha, mitochondrial	0.57	<0.001
P83884	*RPL36*	60S ribosomal protein L36	0.60	0.012
A5GFQ0	*RPL7*	60S ribosomal protein L7	0.60	0.004
F1RQ90	*RBMX*	RNA-binding motif protein, X chromosome isoform 1	0.65	<0.001
F1S2 × 3	*ECHDC1*	Ethylmalonyl-CoA decarboxylase 1	1.53	0.049
A0A287ARZ1	*TXNDC5*	Thioredoxin domain-containing protein 5	1.73	0.020
A0A287A6U0	*NRGN*	Neurogranin	2.22	0.003

## Data Availability

The MS proteomics data has been uploaded to the ProteomeXchange Consortium via the PRIDE partner repository with the dataset identifier PXD033684.

## Supplementary Materials

The following are available online at https://www.mdpi.com/article/10.3390/antiox11061135/s1, Table S1: Statistical results of the GO overrepresentation test of the comparison between the Ctrl and HTX groups.
