# Supplementary material for: Polyphenols and IUGR Pregnancies: Effects of the Antioxidant Hydroxytyrosol on the Hippocampus Proteome in a Porcine Model
Source: Antioxidants (Basel). 2022 Jun 9;11(6):1135. doi: 10.3390/antiox11061135 (PMC9219860; doi:10.3390/antiox11061135)
Supplement: Supplementary file 1 [file antioxidants-11-01135-s001.zip › antioxidants-1737390-supplementary.pdf]

**Supplementary Table S1:** Statistical results of the GO overrepresentation test of the comparison between the Ctrl and HTX groups

**GO molecular function complete**

| GO molecular function complete | # | # | expected | fold enrichment | +/− | raw *p*-value | FDR |
|---|---|---|---|---|---|---|---|
| enoyl-CoA hydratase activity | 10 | 2 | .01 | > 100 | + | $1.71 \times 10^{-5}$ | $2.79 \times 10^{-2}$ |
| structural constituent of ribosome | 168 | 4 | .09 | 44.56 | + | $1.48\times 10^{-6}$ | $7.26 \times 10^{-3}$ |
| RNA binding | 1655 | 7 | .88 | 7.92 | + | $5.42\times 10^{-6}$ | $1.33 \times 10^{-2}$ |

**GO biological process complete**

| GO biological process complete | # | # | expected | fold enrichment | +/− | raw *p*-value | FDR |
|---|---|---|---|---|---|---|---|
| cytoplasmic translation | 124 | 4 | .07 | 60.38 | + | $4.54 \times 10^{-7}$ | $7.11 \times 10^{-3}$ |
| translation | 377 | 5 | .20 | 24.82 | + | $9.00 \times 10^{-7}$ | $7.06 \times 10^{-3}$ |
| peptide biosynthetic process | 404 | 5 | .22 | 23.16 | + | $1.26 \times 10^{-6}$ | $6.59 \times 10^{-3}$ |
| peptide metabolic process | 538 | 5 | .29 | 17.40 | + | $5.06 \times 10^{-6}$ | $1.59 \times 10^{-2}$ |
| amide biosynthetic process | 520 | 5 | .28 | 18.00 | + | $4.29 \times 10^{-6}$ | $1.68 \times 10^{-2}$ |

**GO cellular component complete**

| GO cellular component complete | # | # | expected | fold enrichment | +/− | raw *p*-value | FDR |
|---|---|---|---|---|---|---|---|
| polysomal ribosome | 32 | 2 | .02 | > 100 | + | $1.44 \times 10^{-4}$ | $4.20 \times 10^{-2}$ |
| ribosome | 226 | 4 | .12 | 33.13 | + | $4.70 \times 10^{-6}$ | $2.40 \times 10^{-3}$ |
| ribonucleoprotein complex | 687 | 5 | .37 | 13.62 | + | $1.65 \times 10^{-5}$ | $6.72 \times 10^{-3}$ |
| cytosolic large ribosomal subunit | 58 | 3 | .03 | 96.81 | + | $4.01 \times 10^{-6}$ | $2.73 \times 10^{-3}$ |
| cytosolic ribosome | 104 | 4 | .06 | 71.99 | + | $2.29 \times 10^{-7}$ | $4.68 \times 10^{-4}$ |
| large ribosomal subunit | 113 | 3 | .06 | 49.69 | + | $2.78 \times 10^{-5}$ | $9.45 \times 10^{-3}$ |
| ribosomal subunit | 187 | 4 | .10 | 40.04 | + | $2.25 \times 10^{-6}$ | $2.29 \times 10^{-3}$ |
